# A-T_Winnipeg_: Pathogenesis of rare *ATM* missense mutation c.6200C>A with decreased protein expression and downstream signaling, early-onset dystonia, cancer, and life-threatening radiotoxicity

**DOI:** 10.1002/mgg3.72

**Published:** 2014-03-13

**Authors:** Kotoka Nakamura, Francesca Fike, Sara Haghayegh, Rachel Saunders-Pullman, Angelika J Dawson, Thilo Dörk, Richard A Gatti

**Affiliations:** 1 Department of Pathology and Laboratory Medicine, UCLA School of MedicineLos Angeles, California; 2 Department of Neurology, Albert Einstein College of MedicineBronx, New York; 3 Cytogenetics Laboratory, Division of Laboratory Medicine & Pathology, Departments of Biochemistry & Medical Genetics and Pediatrics & Child Health, Diagnostic Services of Manitoba, University of ManitobaWinnipeg, Manitoba, Canada; 4 Gynaecology Research Unit, Hannover Medical SchoolHannover, Germany; 5 Department of Human Genetics, UCLA School of MedicineLos Angeles, California; 6 Molecular Biology Institute, UCLALos Angeles, California

**Keywords:** Ataxia, ATM, cancer susceptibility, cDNA sequencing, dystonia, missense mutation, telangiectasia, radiosensitivity

## Abstract

We studied 10 Mennonite patients who carry the c.6200C>A missense mutation (p.A2067D) in the *ATM* gene, all of whom exhibited a phenotypic variant of ataxia-telangiectasia (A-T) that is characterized by early-onset dystonia and late-onset mild ataxia, as previously described. This report provides the pathogenetic evidence for this mutation on cellular functions. Several patients have developed cancer and subsequently experienced life-threatening adverse reactions to radiation (radiotoxicity) and/or chemotherapy. As the c.6200C>A mutation is, thus far, unique to the Mennonite population and is always associated with the same haplotype or haplovariant, it was important to rule out any possible confounding DNA variant on the same haplotype. Lymphoblastoid cells derived from Mennonite patients expressed small amounts of ATM protein, which had no autophosphorylation activity at ATM Ser1981, and trace-to-absent transphosphorylation of downstream ATM targets. A-T lymphoblastoid cells stably transfected with ATM cDNA which had been mutated for c.6200C>A did not show a detectable amount of ATM protein. The same stable cell line with mutated *ATM* cDNA also showed a trace-to-absent transphosphorylation of downstream ATM targets SMC1pSer966 and KAP1pSer824. From these results, we conclude that c.6200A is the disease-causing *ATM* mutation on this haplotype. The presence of at least trace amounts of ATM kinase activity on some immunoblots may account for the late-onset, mild ataxia of these patients. The cause of the dystonia remains unclear. Because this dystonia-ataxia phenotype is often encountered in the Mennonite population in association with cancer and adverse reactions to chemotherapy, an early diagnosis is important.

## Introduction

Ataxia-Telangiectasia (A-T) is a rare autosomal recessive genetic disorder caused by mutations in the *ATM* (A-T mutated) gene (OMIM# 607585). Patients with A-T typically demonstrate early-onset ataxia, ocular apraxia, and dysarthria and progressive cerebellar degeneration with later telangiectasia, cancer, and immunodeficiency. Laboratory testing reveals an elevated serum alpha-fetoprotein (AFP), sensitivity to ionizing radiation (IR) by colony survival assay, chromosomal translocations, and cell cycle abnormalities (Boder and Sedgwick 1958; Woods and Taylor 1992; Gatti 2001; Sun et al. 2002). We have studied a mutation c.6200C>A (p.A2067D) in the *ATM* gene that is unique to the Mennonite populations of Canada, Mexico, Central America, Northern Germany, and Netherlands, and is usually associated with dystonia, not ataxia, in young patients (Sandoval et al. 1999; Yanofsky et al. 2009; Saunders-Pullman et al. 2012). The c.6200C>A mutation also appears to be a strong predictor for cancer susceptibility and adverse reactions to radiation or chemotherapy.

Most North American A-T patients inherit different mutations from each parent, that is, they are compound heterozygotes. Approximately 90% of these mutations are either indels, nonsense, or splicing types, resulting mainly in frameshifts and premature termination codons with unstable or undetectable ATM protein. Approximately 10% of conventional A-T patients have bonafide missense mutations (http://www.LOVD.nl/ATM). That said, it is usually difficult to determine whether a missense change is disease-causing or represents a variant of no biological significance unless the DNA/RNA change is transfected into an A-T cell to determine whether it abrogates the ATM protein deficiency and cellular phenotype (Zhang et al. 1997; Mitui et al. 2009). For any disease-causing mutation that is associated with an unusual A-T phenotype, it becomes especially important to determine whether a second nonobvious change *on the same haplotype* might be the true disease-causing variant. Herein, we provide evidence that c.6200C>A is the disease-causing mutation, carried on a unique *ATM* haplotype and associated with an unusual phenotype of early-onset dystonia (i.e., A-T_Winnipeg_).

## Material and Methods

### Cell lines and media

Lymphoblastoid cell lines (LCL) were derived from peripheral blood lymphocytes and were maintained in RPMI media with 10% FBS and 1%PSG. The cells were expanded for clinical testing of ATM status and studied under IRB-approved protocols. The patients were from Canada, Mexico, Belize, United States, and Northern Germany (formerly East Frisia) and all but the latter were Mennonites.

### Mutation analysis

Lymphoblastoid cell lines were treated with cycloheximide for 6 h before isolating total RNA, using RNeasy (Qiagen, Valencia, CA); cDNA was synthesized using random primers and the Superscript III reverse transcriptase (Invitrogen, Carlsbad, CA). The entire *ATM* coding region was divided into eight overlapping fragments (Regions 1–8) ranging from 1500 to 1800 bps (Telatar et al. 1996; Du et al. 2008; Nakamura et al. 2011). These regions were PCR-amplified and then sequenced using 19 different primer sets. All variants at the cDNA level were subsequently confirmed on genomic DNA by sequencing from relevant exon/intron boundaries (see below). Mutation analysis is based on the same ATM reference sequence used for ATM mutations in the Leiden Open Variation Database (http://www.LOVD.nl/ATM) (GeneBank reference sequence:NM_000051.3).

### Real-time PCR

The cDNA was synthesized using qScript cDNA supermix (Quanta Biosciences, Gaithersburg, MD) using manufacturer's protocols. Briefly, 1 µg of total RNA was used as template and mixed with 4 µL of qScript cDNA supermix (5X) and H_2_O to a total volume of 20 *μ*L in 0.2 mL microtube and incubated 5 min at 25°C, 30 min at 42°C, 5 min at 85°C, and held at 4°C. After completion of cDNA synthesis, a 1/20 dilution was used for PCR amplification. Real-Time quantitative PCR for *ATM* mRNA expression was performed using Perfecta SYBR Green FastMix (Quanta Biosciences), as described in manufacturer's protocol. *GAPDH* was used as an internal control to normalize *ATM* mRNA levels.

### STR and SNP haplotype analysis

Standardized STR (Short Tandem Repeat/microsatellite) genotyping for the *ATM* gene region was performed as previously described (Mitui et al. 2003). Briefly, we used four fluorescently labeled microsatellite markers located within a 1.4 cM region of chromosome 11q22-23: D11S1819, NS22, D11S2179, and D11S1818 (Mitui et al. 2003). Markers NS22 and D11S2179 are located within the *ATM* gene, in introns 45 and 62, respectively (Vanagaite, et al., 1995; Udar et al. 1999). Allelic sizes were detected with an ABI 3730 DNA analyzer and standardized to a reference sample (CEPH1347-02). SNP haplotyping was performed using three major polymorphic sites (IVS17-56G>A, 5557G>A, and IVS62-55T>C) as previously described (Thorstenson et al. 2001; Campbell et al. 2003; Mitui et al. 2003).

### Western blotting

Nuclear extracts were prepared by following the NE-PER protocol (Thermo Fisher Scientific, Rockford, IL). Proteins were separated on a 7.5% SDS-polyacrylamide gel. Western blots were prepared as described and probed with anti-ATM (Novus Biologicals, Littleton, CO), anti-phosphoATM (pSer1981), anti-p53 (Cell Signaling, Danvers, MA), anti-KAP1 (Novus Biologicals), anti-phosphoKAP1 (pSer824) (Novus Biologicals), anti-SMC1 (Novus Biologicals), anti-phosphoSMC1 (pSer966) (Novus Biologicals), anti-p84 (GeneTex, Irvine, CA), and anti-GAPDH (GeneTex).

### SIFT and PolyPhen analysis

The effects of missense substitutions on ATM structure and function were evaluated using the programs PolyPhen-2 (http://genetics.bwh.harvard.edu/pph2/dokuwiki/start) (Adzhubei et al. 2010) and SIFT (http://sift.bii.a-star.edu.sg/) (Ng and Henikoff 2001).

### Site-directed mutagenesis and transfection

To introduce the c.6200A variant into the full-length *ATM* cDNA plasmid construct, pMAT1, we used QuickChangeTM LX site-directed Mutagenesis Kit (Stratagene, La Jolla, CA, USA), according to the manufacturer's protocol and previous protocols (Zhang et al. 1997; Mitui et al. 2009). Ten million AT7LA cells were transfected with 15 *μ*g of mutagenized construct using electroporation (250 V, 1,180 mF). Selection of resistant cells began at 72 h after transfection with 200 *μ*g/mL Hygromycin B (Roche Applied Science, Indianapolis, IN). Stably transfected cells were treated with 7 *μ*mol/L CdCl_2_ for 17 h to induce ATM expression.

### MG-132 exposure

Proteasome inhibitor MG-132 (Calbiochem, San Diego, CA) was dissolved in DMSO. LCLs were treated with 10 *μ*mol/L MG-132 for 4 h before nuclear lysates were prepared for immunoblotting and developed with antibody against ATM protein. To confirm the MG-132 activity, antibody against p53 (Cell Signaling) was used as a control.

## Results

### Mutation and haplotype analyses

**Figure 1:**
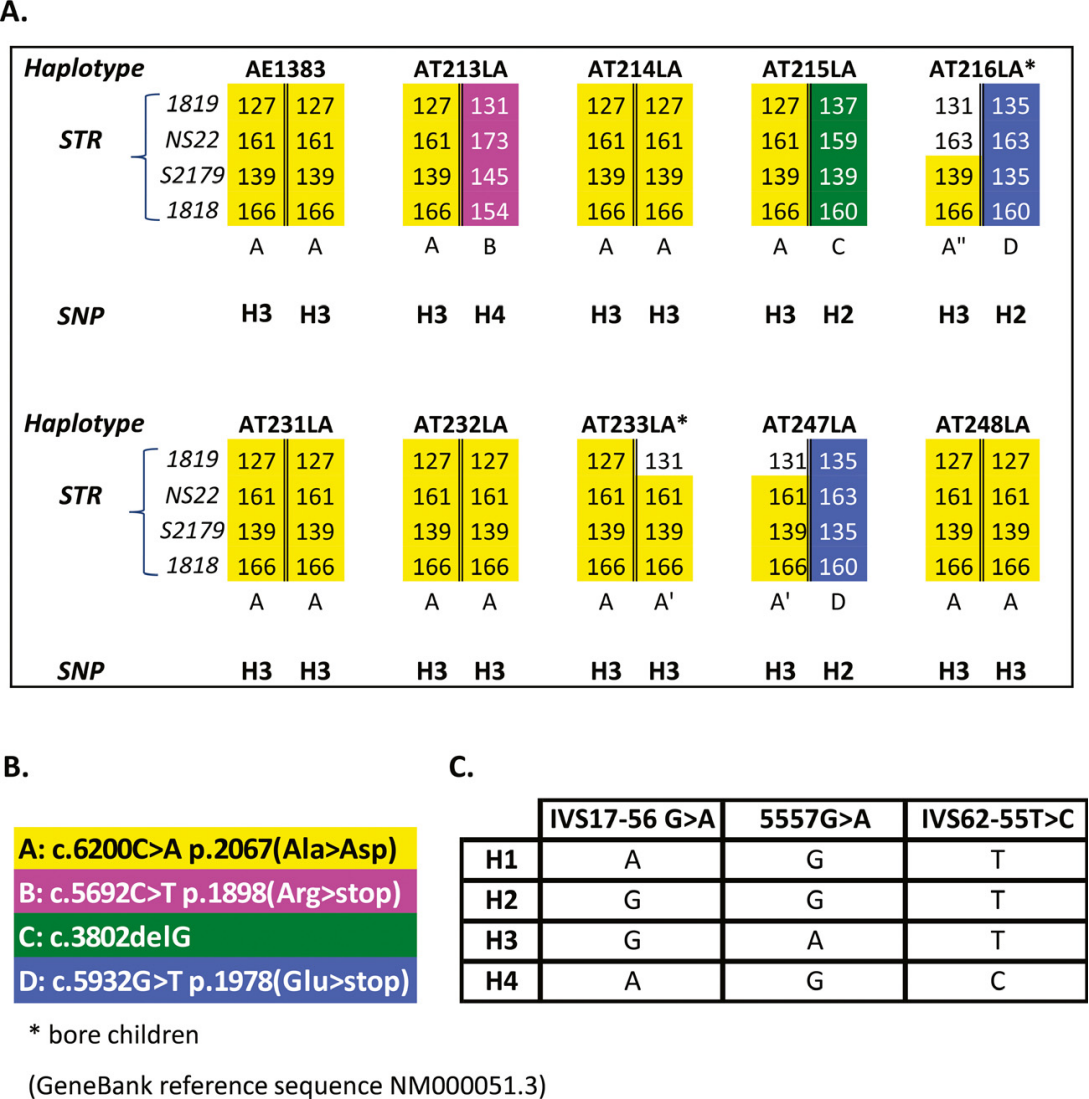
(A) Short Tandem Repeat and SNP haplotypes of 10 Mennonite patients and (B) accompanying mutations. (C) All c.6200C>A mutations were associated with the same SNP haplotype H3 and a unique STR haplotype (or haplovariants).

We sequenced the *ATM* coding region using mRNA isolated from LCLs derived from ten ataxia-telangiectasia patients of Mennonite ethnicity and identified the missense variant c.6200C>A (p.A2067D) in each. As depicted in Figure 1, six were homozygous (AE1383, AT214LA, AT231LA, AT232LA, AT233LA, and AT248LA) and four were compound heterozygotes (AT213LA, AT215LA, AT216LA, and AT247LA). Haplotyping around a particular gene often provides insights into the ethnic ancestry of the mutation carried by that haplotype. The STR (Short Tandem Repeat) haplotype associated with the c.6200C>A variant was identified as: (S1819, *127*; NS22, *161*; S2179, *139*; and S1818, *166*) and segregated in trans in AT213LA, AT232LA, and AT233LA. Some patients (AT216LA, AT233LA, and AT247LA) carried only partial variations of this haplotype (i.e., haplovariants): (A′ and A″) (Fig. 1). Patients AT216LA and AT247LA also carried a c.5932G>T mutation on haplotype D (S1819, *135*; NS22, *163*; S2179, *135*; S1818, *160*) (see Fig. 1), a mutation/haplotype common in Polish and Russian populations (Telatar et al. 1998; Birrell et al. 2005; Mitui et al. 2005). The c.6200C>A variant was always associated with an H3 SNP haplotype and the unique STR haplotype (Fig. 1). Both SNP and STR haplotypes are characteristic for Europeans (Thorstenson et al. 2001; Campbell et al. 2003). A previously reported German patient, Ae003 (Sandoval et al. 1999) with the c.6200C>A mutation, had the same STR haplotype A and SNP haplotype H3, as shown in Figure 1(Ae003 is not shown), which segregated in trans. [The clinical features of patients associated with LCLs AT213LA, AT214LA, and AT215LA have been previously described by Yanofsky et al. (2009) as Patients 1, 2, and 3, respectively. The neurological features of patients associated with AT231LA and AE1383 have been previously described by Saunders-Pullman et al. (2012) as Cases C:301 and C:302, respectively.]

### Detection of ATM protein and kinase activities

**Figure 2:**
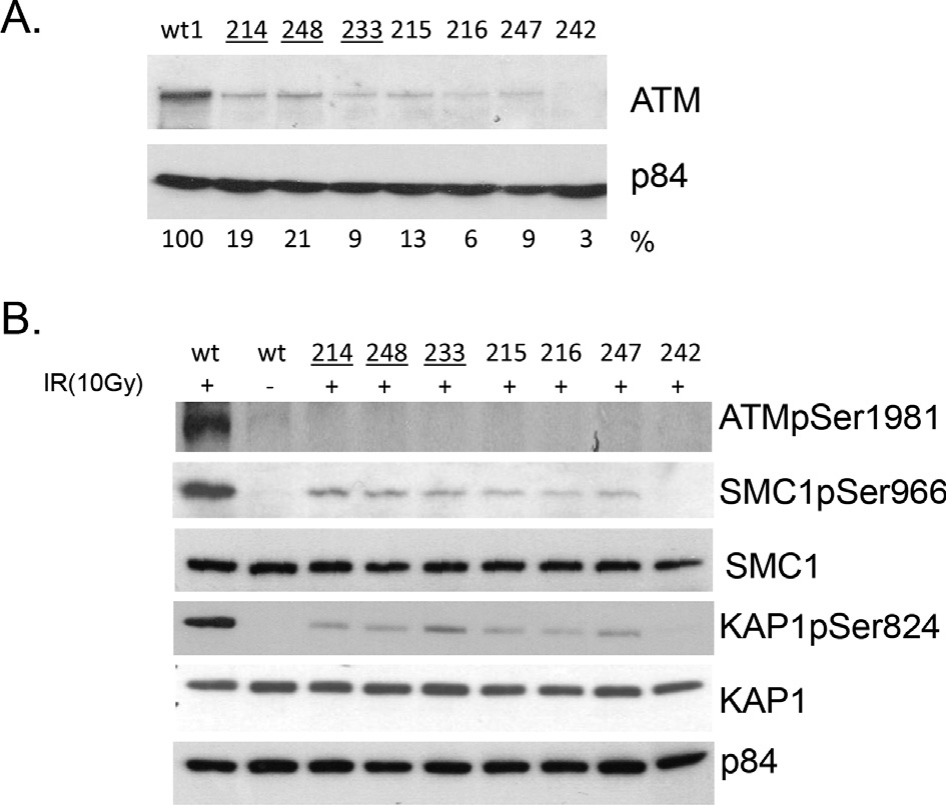
(A) Western blots showed small amounts of ATM protein in homozygous (lanes 2–4) and heterozygous (lanes 5–7) patients in contrast to the absence of protein in a typical A-T patient like AT242LA with two nonsense mutations (lane 8). Nuclear extracts were isolated from LCLs. P84 was used as a loading control. Cells from patients homozygous for the c.6200C>A mutation are underlined. Quantification of ATM protein was done with Quantity One (Biorad) (bottom row). (B) ATM kinase activity and phosphorylation of the downstream target proteins. Cell extracts were prepared 45 min after 10 Gy irradiation. ATMpSer1981 was used to detect autophosphorylation of ATM protein. Native SMC1, KAP1 and p84 were used as loading controls.

ATM protein was measured in LCLs from six patients: three were homozygous (AT214LA, AT248LA, AT233LA) (Fig. 2A; lanes 2, 3 and 4) and three were heterozygous (AT215LA, AT216LA, AT247LA) (Fig. 2A; lanes 5, 6 and 7). All nuclear extracts showed trace amounts of ATM, except for AT242LA (Fig. 2A; lane 8), which had two unrelated (i.e., not 6200C>A) nonsense mutations and showed no ATM protein. The wt (wild type) cell line (Fig. 2A; lane 1) was used as the normal control for ATM protein level. From previous experience (Chun et al. 2003), ATM levels from nuclear lysates of A-T cell lines typically show less than 15% of wild-type cell levels, unless a missense mutation is present (∼10% of conventional A-T patients), in which case the ATM level is sometimes higher (Stankovic et al. 1998); but with variable kinase activity; the colony survival level is typically in the radiosensitive range for A-T patients (Sun et al. 2002).

To assess ATM kinase activity, LCLs were exposed to 10 Gy ionizing radiation and harvested 45 min after IR damage. While all A-T samples showed no detectable ATM pSer1981 autophosphorylation, we observed weak transphosphorylation activity of downstream ATM targets, SMC1pSer966 and KAP1pSer824 (Fig. 2B, lanes 3–8). As anticipated, an A-T cell line with two unrelated nonsense mutations, AT242LA, showed no ATM-dependent kinase activity (lane 9). The irradiated WT (wild type) cell line showed normal post-10 Gy ATM kinase levels for SMC1 and KAP1 (Fig. 2B, lane 1).

### Measurement of nonsense-mediated mRNA decay

**Figure 3:**
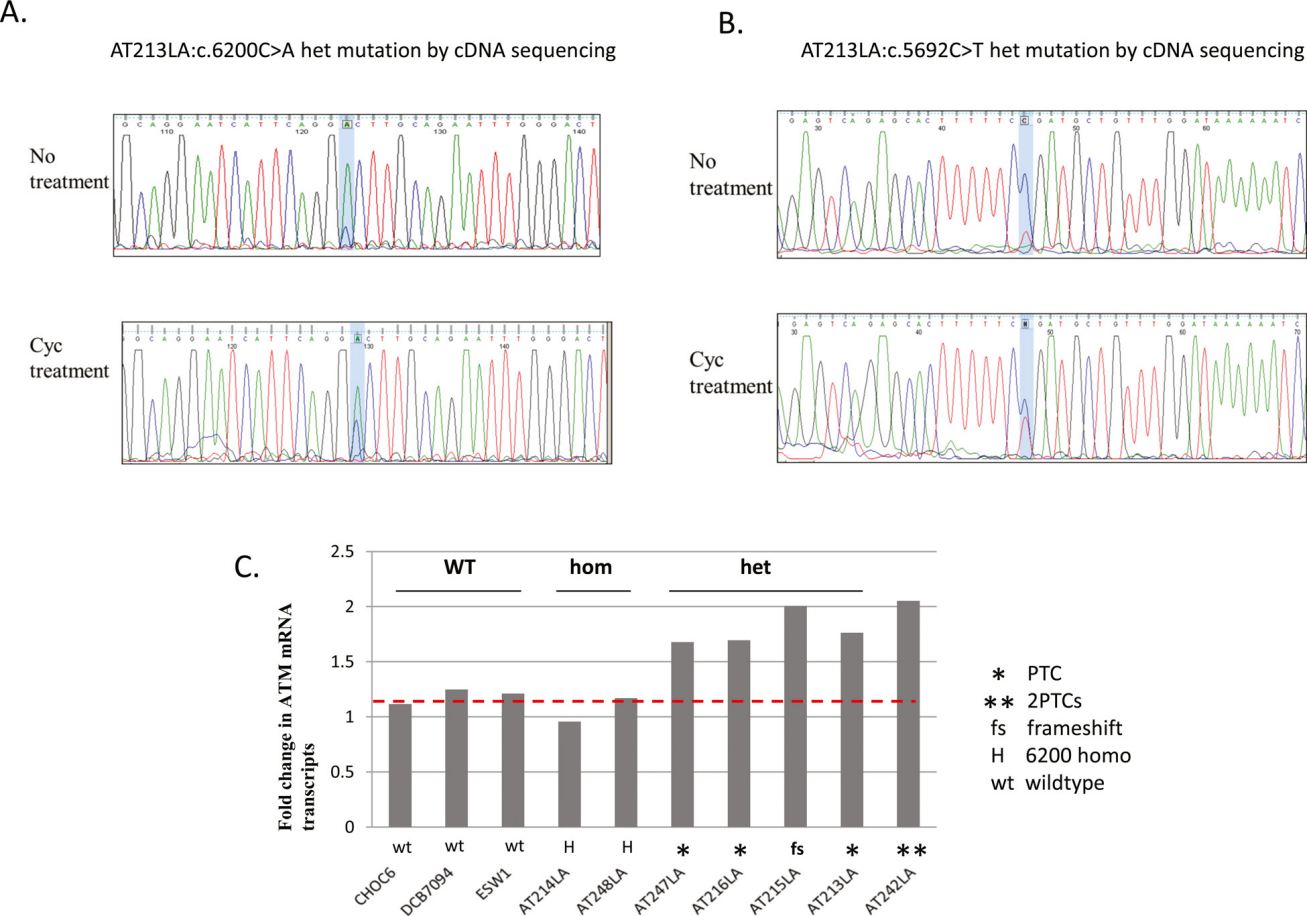
(A) and (B) two sets of incongruous DNA sequencing chromatographs for two mutations in AT213LA cells. Note that RNA sequences differed after cells were grown in cycloheximide (blue shading). Without cycloheximide, both mutations appear to be homozygous, whereas after cycloheximide inhibition of NMD, sequences suggest heterozygosity, which was confirmed by genomic DNA sequencing and supported by haplotyping (see Fig. 1). (C) RT-PCR results showed increased *ATM* mRNA levels after cycloheximide treatment in four patients carrying nonsense mutations (AT247LA, AT216LA, AT215LA, AT213LA). CHOC6, DCB7094, and ESW1 are wild type. AT214LA and AT248LA are homozygous for the 6200C>A missense mutation. AT242LA derives from an A-T patient with two other nonsense mutations. The levels of expression in the treated cells were normalized to the levels of the paired untreated cells, so as to compare the relative effects after cycloheximide treatment. Data are mean ± SD of triplicate.

In three patients, initial cDNA sequencing showed 6200C>A as an apparently homozygous change (Fig. 3A top). Despite this, when confirming the mutation by genomic DNA (gDNA), heterozygosity was realized. To investigate the possible activity of nonsense-mediated mRNA decay (NMD), we treated LCLs with cycloheximide prior to isolating total RNA and sequencing. This RNA showed the c.6200C>A variant as a heterozygous change (Fig. 3A, bottom) and we were subsequently able to identify the second mutation in AT213LA as c.5692C>T (Fig. 3B bottom sequencing panel). Second mutations were similarly identified in AT216LA (c.5932G>T), AT247LA (c.5932G>T), and AT215LA (c.3802delG), as depicted in Figure 1.

To further demonstrate the effects of NMD on the *ATM* mRNA, we used RT-PCR with or without cycloheximide treatment of the cells to study six Mennonite patients: two were homozygous for c.6200C>A (AT214LA; AT248LA), three with accompanying nonsense (PTC) mutations (AT247LA, AT216LA, andAT213LA), and one with an accompanying frameshifting mutation (AT215LA), which causes a secondary downstream premature termination codon (PTC). We also used three wild-type cells as controls and a non-Mennonite LCL, AT242LA, carrying two nonsense mutations (see above text and legend of Fig. 2). As shown in Figure 3C, wild-type cells (CHOC6, DCB7094, and ESW1) and the two homozygous c.6200C>A missense variants (AT214LA and AT248LA) did not show significant changes in *ATM* mRNA level before and after treatment with cycloheximide, as compared to the four cells with nonsense (PTC) mutations (AT247LA, AT216LA, AT213LA, and AT242LA) whose mRNA levels increased approximately twofold after treatment. The cycloheximide-associated increase in mRNA levels observed in AT215LA may be due to a secondary PTC codon created by a frameshifting delG mutation. Further inspection of the cDNA sequencing data failed to identify a third *ATM* mutation.

### Polyphen and SIFT analysis for p.A2067D variant

The Polyphen-2 (*Poly*morphism *Phen*otyping) program evaluates the possible impact of an amino acid substitution on the structure and function of a human protein (Adzhubei et al. 2010). In this case, it predicts that the substitution of an Alanine (A) with an Aspartate (D) at position 2067 is “probably damaging”, returning a hypothetical score of 1.000 and 0.974 by the HumDiv and HumVar databases, respectively. Multiple alignments of the area of substitution showed strong conservation throughout modern evolution, lending further support to the interpretation that the p.A2067D variant is pathogenic (Suppl. Fig. S2a). Moreover, the SIFT algorithms, which predict the functional importance of amino acid substitutions based on the alignment of orthologous and/or paralogous protein sequences, predicted that the substitution of the nonpolar alanine (A) with an acidic aspartate (D) would fall into the “Not Tolerated” zone (Suppl. Fig. S2b).

### Site-directed mutagenesis to introduce c.6200A variant

**Figure 4:**
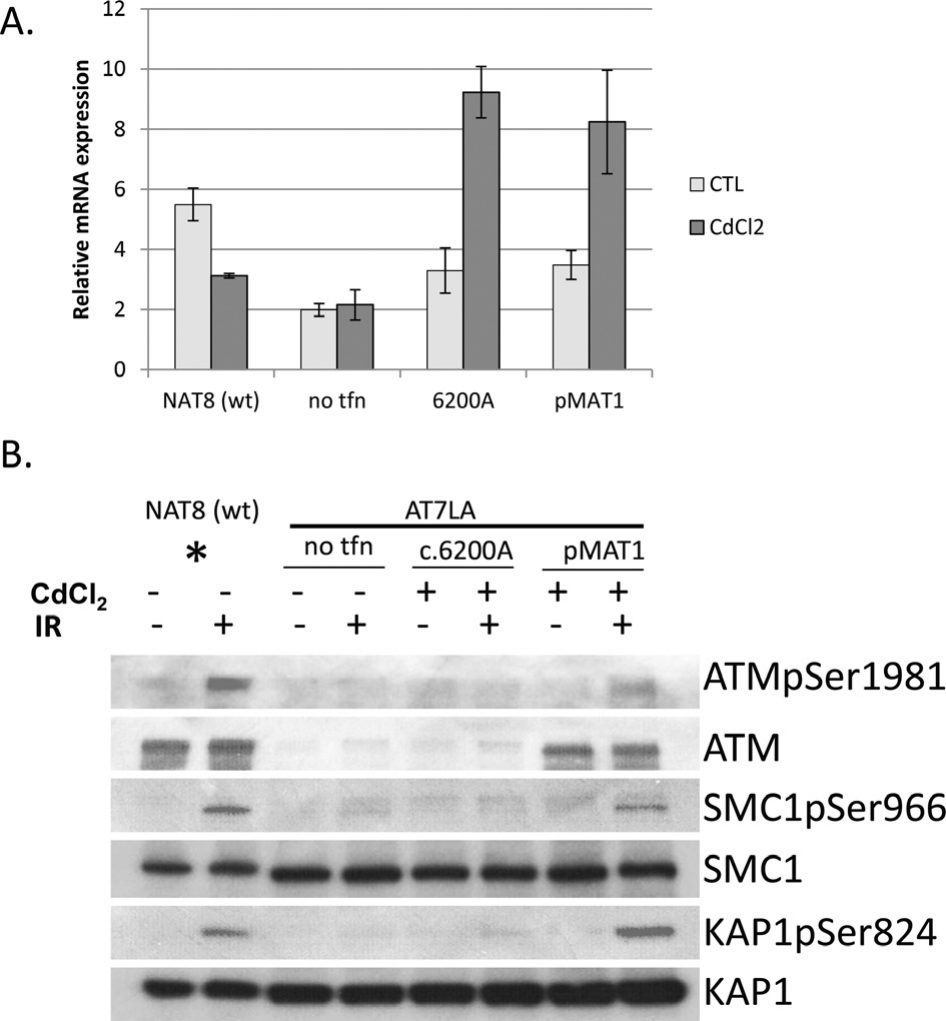
Transfection of c.6200A mutation into A-T LCL (AT7LA) showed trace-to-absent ATM protein or kinase function. (A). WT, AT7LA, AT7LA-stably transfected with pMAT1 with c.6200C>A mutation (c.6200A), and AT7LA-stably transfected with full-length wild-type *ATM* cDNA-expression vector pMAT1, were treated with 7 *μ*mol/L CdCl_2_ for 17 h; RNAs were then isolated to analyze the change in the levels of ATM transcripts by RT-QPCR normalized to *GAPDH* transcripts. Data are mean ± SD of triplicate. (B). After 17 h treatment with 7 *μ*mol/L CdCl_2_, cells were irradiated with 10 Gy and lysed 45 min after irradiation. 50 *μ*g of nuclear lysates were loaded for AT7LA and transfectants. Native KAP1 and SMC1 were also utilized as loading controls. The asterisk denotes that for the NAT8 (wild type) controls only 25 *μ*g of nuclear lysate was loaded to avoid subsequent overexposure of the autoradiographs.

In order to determine whether the c.6200C>A missense variant is disease-causing or a passenger SNP or polymorphism, we introduced the c.6200A mutation into the pMAT1 vector, containing wild-type full-length ATM cDNA, by site-directed mutagenesis. After inducing the metallothionein II promoter of pMAT1 with cadmium chloride, we observed that the ATM transcript levels increased for AT7LA cells stably transfected with either the pMAT1 vector containing 6200C>A or the unadulterated pMAT1 (Fig. 4A), confirming success of stable transfection and induction of the transfected ATM gene. The same cells were irradiated with 10 Gy to create double strand DNA break damage and were harvested 45 min thereafter to analyze ATM protein expression and its kinase activity by immunoblotting (Zhang et al. 1997; Mitui et al. 2009). As shown in Figure 4B, lanes 7 and 8, AT7LA (A-T cells) with pMAT1 construct expressed ATM protein and showed functional kinase activities of: (1) autophosphorylation of ATM itself (ATMpSer1981) and (2) downstream targets SMC1pSer966 and KAP1pSer824 after irradiation. However, in AT7LA cells transfected with the 6200A mutation construct, ATM protein was not detectable (Fig. 4B, lanes 5 and 6) and there was a trace-or-absent signal for phosphorylation of ATMpSer1981, SMC1pSer966, or KAP1pSer824 in the irradiated samples (indicated by “+” in Fig. 4B). As expected, no endogenous ATM protein was observed in uninduced AT7LA cells stably transfected with pMAT1 or pMAT1 with 6200A mutation (Fig. S1, Lanes 3–8). These cells also failed to show autophosphorylation of ATM and kinase activities for downstream targets after the irradiation (lanes 4, 6, and 8). [For wild-type LCL (NAT8) (lanes 1 and 2) only 50% of protein was loaded to avoid overexposure of ATM as indicated by an asterisk].

### Protein stability and proteasome inhibition by MG132

**Figure 5:**
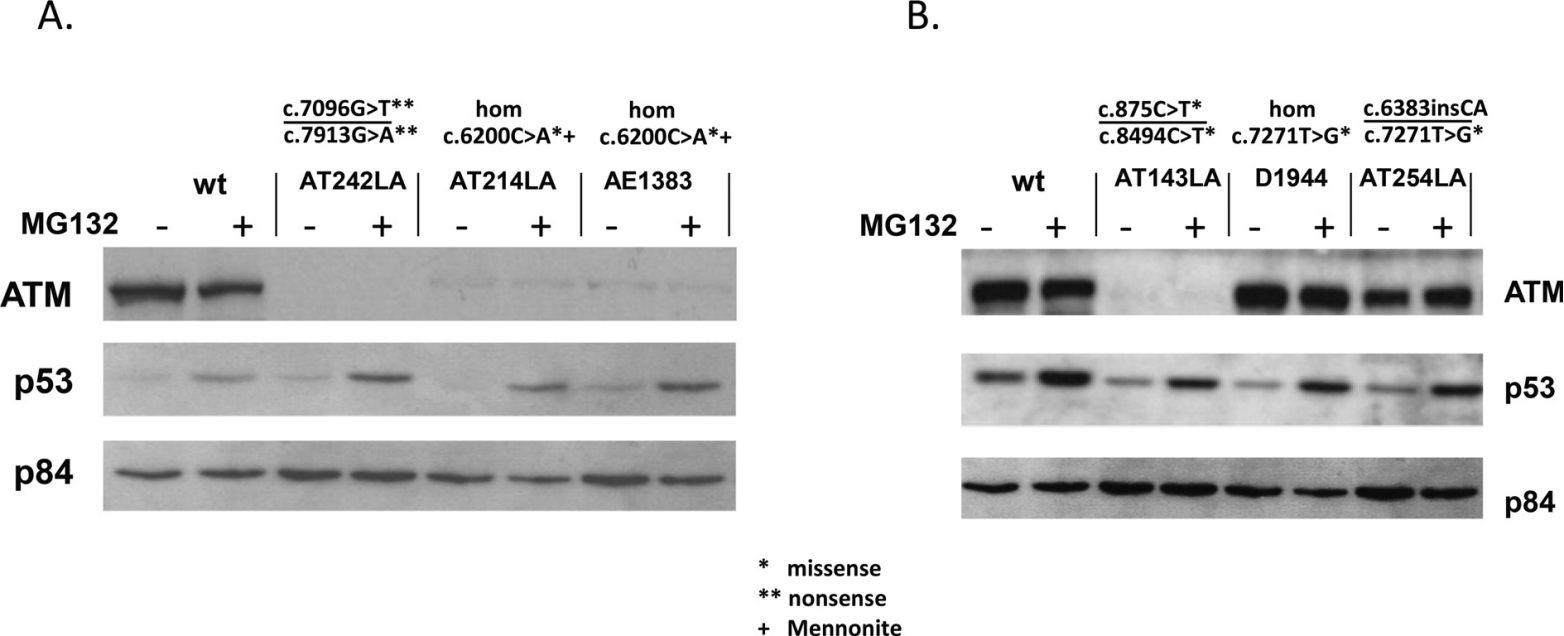
Immunoblots showing that ATM 6200A protein is not a target for proteasome degradation. (A) Treatment of cells with the proteasome inhibitor MG-132 for 4 h did not increase the level of ATM protein. AT242LA is an A-T patient with two other nonsense mutations; AT214LA and AE1383 are Mennonite A-T patients who are homozygous for c.6200C>A. (B) AT143LA, D1944 ,and AT254LA are LCLs from patients with non-Mennonite missense mutations. MG-132 did not increase the levels of ATM protein. P53 was used as an MG-132 activity control and p84 was used as a loading control.

As discussed above, a pMAT1 construct carrying the c.6200A variant showed no ATM protein after CdCl_2_ induction (Fig. 4B, lane 5) and reduced ATM protein in patients' cells (Fig. 2A lanes 2–7) suggesting that either its production was reduced or the variant protein may be targeted by the proteasome for degradation (Maki et al. 1996). To test the latter possibility, we treated patient-derived LCLs that were homozygous for 6200C>A (AE1383 and AT214LA) with the proteasome inhibitor MG132. No increase was observed in ATM protein levels after proteasome inhibition (Fig. 5A). As a positive control, MG132-treated cells (+) showed increased p53 protein levels; p53 is known to be targeted by proteasome degradation (Maki et al. 1996). We also treated cells from non-Mennonite patients with other missense mutations (AT143LA, D1944 and AT254LA) with MG132 (Fig. 5B). AT143LA cells showed no detectable ATM protein before or after treatment with MG132. D1944 and AT254LA cells (both carrying “kinase-dead” mutations) also showed no significant differences in ATM levels after MG132 treatment (D1944 was originally described by Stankovic et al. 1998).

## Discussion

We analyzed 10 patients with mild A-T phenotypes from Mennonite families and identified the c.6200C>A (p.A2067D) missense mutation in all. All patients also manifested dystonia from an early age (Yanofsky et al. 2009; Saunders-Pullman et al. 2012). The onset of ataxia was later than that of this dystonia, especially in the c.6200C>A homozygotes. The cancer risk in this population is high but as yet cannot be formally established because of the limited number of patients. Life-threatening responses to chemotherapy and/or radiation therapy were seen in three patients (Yanofsky et al. 2009). Two patients had borne children.

The laboratory assessment of the c.6200C>A variant was confounded by several factors: (1) patients homozygous for the c.6200C>A variant had more than just trace amounts of ATM protein, as has been previously observed for another cancer-associated missense *ATM* mutation, c.7271T>G. (Stankovic et al. 1998). (2) RNA sequencing *misleadingly* suggested that an additional four patients were homozygous for the c.6200C>A variant (and this was supported by the patients' consanguineous histories). However, sequencing of genomic DNA did not confirm the homozygosity and second accompanying mutations were eventually identified – and haplotyping further supported heterozygosity - when new RNA was collected from LCLs regrown in the presence of cycloheximide to inhibit NMD (see Fig. 3). Ironically, if RNA sequencing were to gain in popularity as an alternative to DNA sequencing, such problems created by NMD would have to be mitigated; (3) because the c.6200C>A mutation is very rare outside the Mennonite community, there is little a priori information to supplement the prediction programs that are used to assess the biological significance of this missense mutation; the variant is scored only as “probably damaging”; (4) the possibility existed that with further testing another variant could/might have been identified in association with the observed STR haplotype, further confounding these analyses. Our finding of reduced *ATM* transcript levels associated with truncating mutations, including the c.3802delG frameshift mutation in AT215LA (Fig. 3B), is consistent with previous observations in LCLs from A-T patients (Hüsing et al. 2000) and with the general observation that NMD is a frequent consequence for mRNAs with even secondary premature termination codons (Kervestin and Jacobson 2012).

As a general rule for *ATM* mutations, patients within the same ethnic group who carry the same variant allele also carry the same SNP and STR haplotypes (Campbell et al. 2003). All patients carrying the c.6200C>A variant showed the same SNP H3 haplotype and a unique STR haplotype (or haplovariant). These STR and SNP haplotypes were also found in a non-Mennonite A-T patient, Ae003 (Sandoval et al. 1999). This patient was from northern Germany (formerly Frisia), the region where Father Menno Simons founded the Mennonite Sect (Neff 1957). Thus, *ATM* haplotyping may provide another testing parameter for dystonia patients.

Missense *ATM* variants often produce some protein, and if it is functional we often define them as “probably tolerated” or benign. Only a few on these *ATM* variants produce near-normal levels of protein and those have little or no kinase activity (Stankovic et al. 1998; Angèle et al. 2003; Barone et al. 2009; Mitui et al. 2009; Verhagen et al. 2012). In this study, we established a stably transfected cell line expressing 6200A transcript and demonstrated that this change caused little or no ATM protein expression, with dramatically decreased kinase activity (Fig. 4B, lane 6). In silico analysis using PolyPhen and SIFT (Supplemental Figure S2) predicted that the c.6200C>A change is “probably damaging” and “not tolerated”. Taken together, these results indicate that the c.6200C>A is a pathogenic variant associated with unstable ATM protein.

Proteasomes degrade unstable or nonfunctional proteins. The proteasome is a multisubunit complex that degrades various cellular proteins, and the degradation in this complex requires a specific signal, that is, attachment of a multiubiquitin chain to the target protein (Ciechanover 1994). Most of the missense mutations in the *ATM* gene produce small but detectable amounts of ATM protein. To investigate possible proteasome degradation, we utilized MG-132 to block a ubiquitination-dependent degradation. However, inhibiting proteasome activity did not change ATM protein levels (Fig. 5). It is still possible, however, that another kind of mechanism might be involved in the degradation of missense ATM protein.

In summary, this study demonstrates the disease-causing consequences of a c.6200C>A missense mutation in the *ATM* gene resulting in a p.A2067D substitution that leads to protein instability and kinase deficiency. The mutation is associated with an early-onset dystonia and cancer risk. Residual levels of ATM protein may explain the relatively mild ataxia phenotype in Mennonite patients. A second mutation is sometimes overlooked (Fig. 3). Recently, two independent studies described certain *ATM* missense mutations in patients that are associated with early-onset dystonia (Charlesworth et al. 2013; Meissner et al. 2013); these patients showed a milder A-T phenotype that was diagnosed at a much later age compared to most typical A-T patients. As the rates of malignancy and the risks associated with exposure to radiation are high for these atypical A-T patients, it is important to identify them as early as possible so that appropriate oncological and neurological support can be instituted.

## Supporting Information

**Figure S1.** Negative control for CdCl_2_ induction of metallothionein II promoter. **Figure S2.** Sequence Alignment and Phylogeny of p.2067 (Alanine).
