# Supplementary material for: A-TWinnipeg: Pathogenesis of rare ATM missense mutation c.6200C>A with decreased protein expression and downstream signaling, early-onset dystonia, cancer, and life-threatening radiotoxicity
Source: Mol Genet Genomic Med. 2014 Mar 13;2(4):332–40. doi: 10.1002/mgg3.72 (PMC4113274; doi:10.1002/mgg3.72)
Supplement: Supplementary file 1 [file mgg30002-0332-SD1.docx]

**Figure S1** Negative control for CdCl_2_ induction of metallothionein II promoter. Stable cells without CdCl_2_ treatment showed neither ATM protein nor ATM kinase activities. Cells were damaged with 10 Gy and lysed 45 min after irradiation. 50 *μ*g of nuclear lysates were loaded for AT7LA and transfectants. Native KAP1 and SMC1 were used as loading controls. For the NAT8 (wild type) control (lanes 1 and 2), only 25 *μ*g of nuclear lysate was loaded as indicated by asterisk.

**Figure S2** Sequence Alignment and Phylogeny of p.2067 (Alanine). (A) Sequence alignment of human ATM orthologs in various species confirm that the p.2067A>D amino acid substitution affects a highly conserved region of the protein (ALQN) and is “probably damaging”. HumDiv and HumVar are defined on the PolyPhen website (see methods). (B) SIFT algorithm predicts the functional importance of the alanine/aspartate substitution based on the alignment of orthologous and/or paralogous protein sequences and predicts that the A to D substitution at the p.2067 position would be “Not Tolerated”.
