# Supplementary figures and images for: A-TWinnipeg: Pathogenesis of rare ATM missense mutation c.6200C>A with decreased protein expression and downstream signaling, early-onset dystonia, cancer, and life-threatening radiotoxicity
Source: Mol Genet Genomic Med. 2014 Mar 13;2(4):332–40. doi: 10.1002/mgg3.72 (PMC4113274; doi:10.1002/mgg3.72)

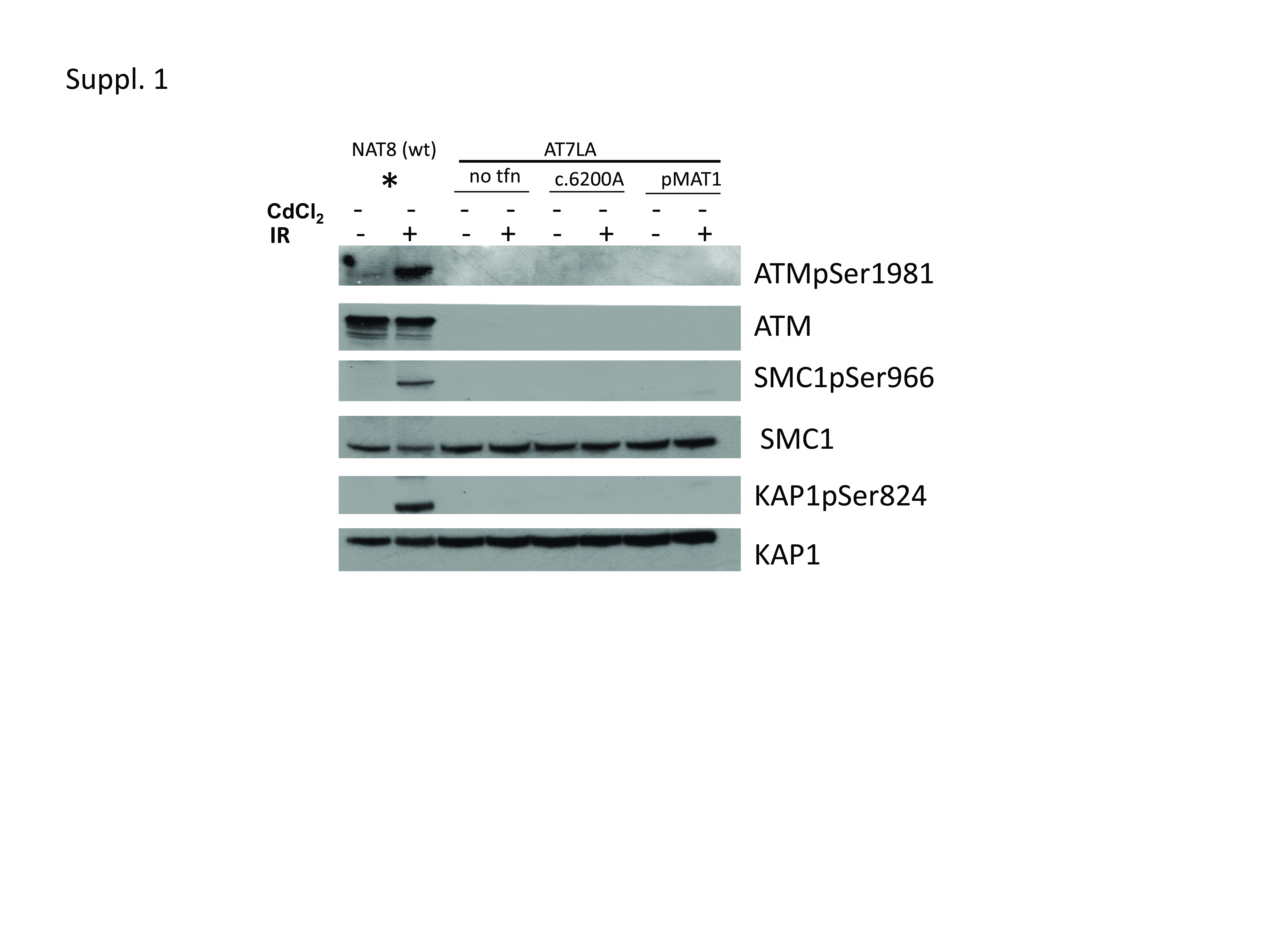

Supplement: Supplementary file 2 [file mgg30002-0332-SD2.tif]

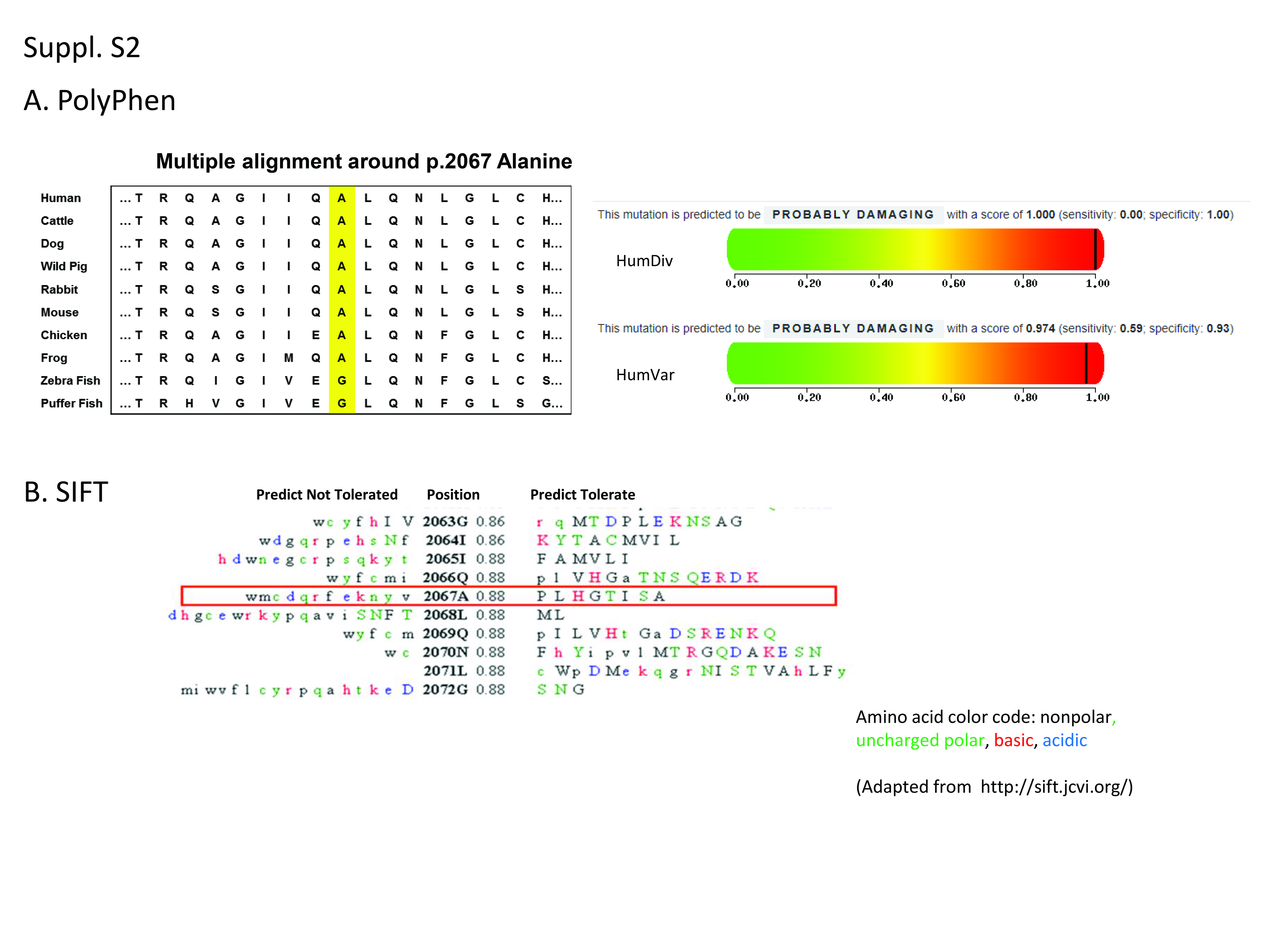

Supplement: Supplementary file 3 [file mgg30002-0332-SD3.tif]
